# Bioinspired Sternal Implant Design for Generic Anatomical Reconstruction: An In Silico Framework for Material Selection and Biomechanical Validation

**DOI:** 10.3390/biomimetics11040251

**Published:** 2026-04-05

**Authors:** Işıl Kutbay, Zeynep Gerdan, Murat Çolak, Yasemin Tabak, Abdullah Tahir Şensoy

**Affiliations:** 1 Department of Electronics and Automation, University of Health Sciences, Uskudar, 34668 Istanbul, Türkiye; isil.kutbay@sbu.edu.tr; 2 Vocational School of Technical Sciences, Department of Digital Health Systems Technician, Bayburt University, 69000 Bayburt, Türkiye; zeynepgerdan@bayburt.edu.tr; 3 Vocational School of Technical Sciences, Department of Electronic Automation, Bayburt University, 69000 Bayburt, Türkiye; mcolak@bayburt.edu.tr; 4 TÜBİTAK National Metrology Institute (UME), 41470 Gebze, Türkiye; yasemin.tabak@tubitak.gov.tr; 5 Faculty of Mechanical Engineering, Delft University of Technology, Mekelweg 2, 2628 CD Delft, The Netherlands; 6 Department of Oral and Maxillofacial Surgery, Erasmus University Medical Center, Doctor Molewaterplein 40, 3015 GE Rotterdam, The Netherlands; 7 Department of Biomedical Engineering, Faculty of Engineering and Natural Sciences, Samsun University, 55420 Samsun, Türkiye

**Keywords:** sternum implant, bioinspired design, CES selector, biomaterial selection, finite element analysis, thoracic reconstruction

## Abstract

The sternum protects the intrathoracic organs and contributes to chest wall mechanics, which makes reconstruction after tumor resection, trauma, or infection a demanding biomechanical problem. This study presents an in silico workflow for preselecting materials for sternal implants before physical prototyping. After a virtual resection, an anatomically conformal implant was designed and candidate biomaterials were screened in CES Selector using density, elastic modulus, fatigue strength, fracture toughness, toxicity, medical grade suitability, and MRI safety. A representative subset of the screened candidates was then compared by finite element modeling in terms of stress transfer and deformation. Seventeen candidates met the screening criteria. Ti-13Nb-13Zr showed an elastic modulus of about 80 GPa, and the titanium-based candidates showed deformation values of about 0.96 to 1.03 mm, whereas GF PEEK reached about 1.74 mm. The stress shielding index also showed that titanium-based materials remained on the implant-dominant side, while polymer-based materials shifted stress transfer toward bone. Taken together, the findings suggest that Ti-13Nb-13Zr offers the best overall balance for load-bearing sternal reconstruction, whereas PEEK-based systems may be more suitable within the present model for hybrid or adjunct designs. The proposed workflow can support early implant planning and guide future experimental and clinical studies.

## 1. Introduction

Material selection is a crucial stage in engineering design because the mechanical performance, durability, and safety of a structure depend directly on the chosen material. This topic has been studied widely in the aerospace [1], automotive [2], energy [3], and biomedical engineering [4] fields. A sound selection process begins with defining the required functional conditions, identifying key properties, comparing realistic candidates, and matching them to the intended use [5,6,7]. Engineering materials, including metals, ceramics, glasses, polymers, elastomers, and composites, offer a broad range of mechanical, physical, and environmental characteristics. As material science has expanded, the selection process has also become more complex because the number of viable alternatives has grown substantially [8,9,10,11,12,13].

In biomedical engineering, where implants and medical devices interact directly with living tissues, material selection becomes even more demanding [4]. For site-specific reconstruction problems such as the sternum, biomechanical role, anatomical location, expected service life, biocompatibility, and imaging considerations all need to be considered together [14,15,16]. Digital tools such as CES help organize material property data and support rapid comparison of competing candidates [13]. Foundational work by Ashby and related materials design frameworks have been especially influential in making material selection more systematic and transparent [6,8,10,13].

The sternum is a flat bone located at the anterior chest wall and plays an important role in protecting thoracic organs, supporting respiratory mechanics, and maintaining thoracic stability [15,16,17]. It is composed of the manubrium, body, and xiphoid process, and like other flat bones it contains a cancellous core surrounded by cortical bone [16]. Because of this anatomy and its mechanical role, reconstructive materials must provide sufficient stability without creating an excessive stiffness mismatch with the surrounding tissue.

Many materials have been explored for sternal and chest wall reconstruction, including titanium alloys, stainless steel systems, polyethylene-based implants, and high-performance polymers such as PEEK [14,17,18,19]. In practice, material choice is guided by stability, biocompatibility, infection risk, manufacturability, radiological behavior, and cosmetic outcome [17,19,20,21]. Polymeric materials attract attention because of their low density, radiolucency, and relatively compliant behavior, but in sternal reconstruction their use is usually considered together with the need to maintain adequate chest wall stability in the central load-bearing region [19,22,23,24,25].

Although the literature includes many reports on sternal reconstruction, implant performance, and clinical technique, studies that focus specifically on systematic biomaterial preselection for the sternum remain limited. Finite element analysis has therefore become an important tool for examining bone–implant interaction, estimating deformation and stress patterns, and refining implant design before fabrication [4,26,27]. Previous work has shown that numerical simulation can improve chest wall reconstruction strategies and support the safer design of advanced sternal implants [19,26,27,28].

The novelty of the present study lies in the establishment of an integrated in silico workflow that combines anatomically conformal implant modeling based on sternum anatomy, CES based material pre-screening, and comparative finite element analysis for early-stage sternal implant development. After virtual resection, the implant geometry was designed in accordance with the anatomical structure, and the candidate materials were subsequently narrowed through systematic screening and biomechanical assessment. The proposed framework is intended to guide pre-experimental decision making rather than to replace clinical evidence.

Therefore, this research aims to establish a bioinspired framework for sternal implant development by considering the boundary conditions of the anterior chest wall. Within this scope, an anatomically conformal implant design workflow was integrated with computer-aided material screening using the CES database and subsequent finite element validation. By evaluating density, Young’s modulus, fatigue strength, fracture toughness, RoHS compliance, toxicity, medical classification, and MRI safety, the study provides a systematic preselection strategy that can reduce early-stage design uncertainty and support surgical planning prior to physical prototyping.

## 2. Materials and Methods

**Figure 1:**
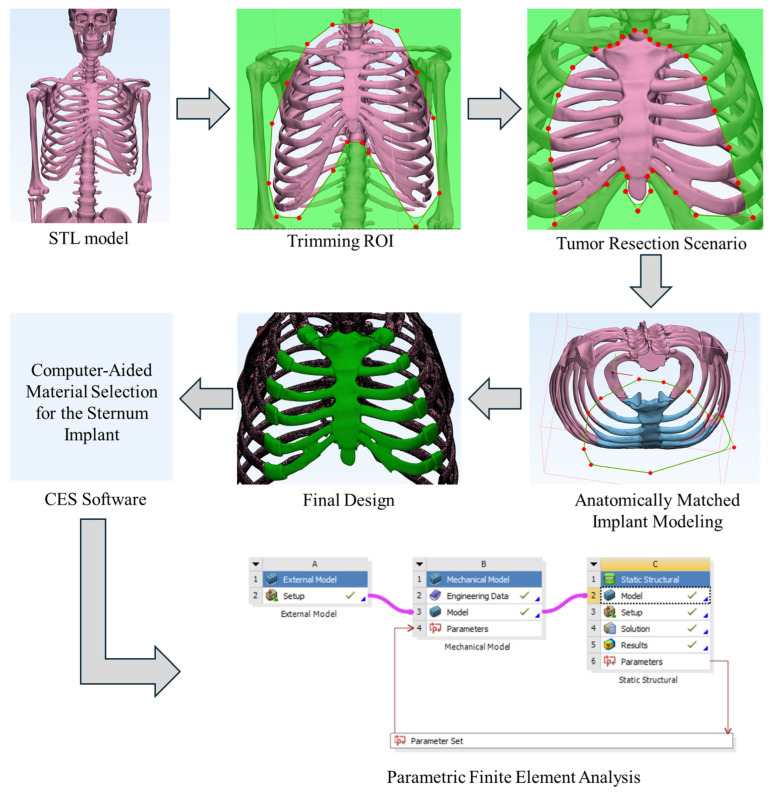
Flowchart of the study.

The methodological flowchart of this study consisted of four main stages: (1) anatomical model acquisition and region of interest (ROI) processing, (2) virtual tumor resection and implant design, (3) multicriteria material selection using the CES Selector database, and (4) biomechanical validation through finite element analysis (FEA). The overall flowchart is illustrated in Figure 1.

### 2.1. Anatomical Modeling

A generic human sternum STL model was initially imported to serve as the anatomical basis for the study. The 3D sternum model, developed by Artec Group Inc., is licensed under the Creative Commons Attribution 3.0 Unported License (CC BY 3.0). The model was processed using Materialise 3-matic 18.0 (Materialise NV, Leuven, Belgium), licensed to Erasmus MC University for academic research.

To reduce geometric complexity and focus computational resources on clinically relevant regions, the region of interest (ROI) was isolated by trimming surrounding structures. Surface artifacts, mesh discontinuities, and irregular manifold features were repaired through local smoothing, remeshing, and edge refinement operations. This preprocessing step ensured topological consistency for the subsequent virtual surgery and implant design stages.

### 2.2. Virtual Surgery

To mimic a realistic clinical condition, the design workflow was initiated with a virtual tumor resection scenario. Although the anatomical basis of the model was generic rather than derived from a patient, the resection-based workflow was intended to emulate the design logic used in patient-adapted reconstruction planning [14,15,17,19]. Using 3-matic surgical simulation tools, a segmental sternectomy was planned virtually. The resection margins were defined based on typical oncological clearance zones reported in thoracic oncology practice.

Following the simulated resection, an anatomically conformal implant was designed using local push-pull and Boolean operations, particularly in the implant and bone connection regions. The implant geometry was shaped to restore the anterior chest wall contour while maintaining attachment compatibility with the rib sternum junctions. All geometries were exported in high-resolution STL format for numerical analysis and mechanical evaluation. This virtual surgical planning workflow provides a reproducible in silico environment for assessing implant performance prior to physical prototyping.

### 2.3. Material Selection

One of the most widely-used computer-aided material selection software tools in materials engineering applications is CES Selector, known for its easy-to-use interface and comprehensive material database. Among its most prominent features are the wide range of materials it offers and its ability to integrate various material selection criteria. The software includes several selection strategies, with the most commonly-used methods being direct database search, use of property comparison charts, filtering based on prescriptive criteria, and multicriteria decision making (MCDM) approaches. Furthermore, Ashby diagrams allow for the visualization of relationships between two material properties and enable the graphical analysis of the positions of different material groups.

In this study, a selection procedure based on predefined threshold values was implemented to determine the appropriate material for a sternum implant. Initially, a suitable material database was selected via the Selection module, followed by the definition of graphical or numerical limit conditions based on mechanical, physical, and thermal properties relevant to the targeted application. Through the software’s Limit function, minimum and maximum values for each property were specified, thereby eliminating materials that did not meet the criteria and generating a list of candidate materials. The database contains extensive information on numerous parameters, including density, elastic modulus, tensile and yield strength, thermal conductivity, and machinability.

During the material selection process, the biomechanical conditions of the anatomical region where the implant is to be used were taken into account. Accordingly, appropriate threshold values were defined for all relevant parameters, and alternative material groups meeting these requirements were identified. The final selection was made by comparing the alternatives within the framework of the specified properties.

A total of 17 candidate biomaterials commonly used in orthopedic and thoracic reconstruction were screened using the CES Selector 2015 (Granta Design Limited, Cambridge, UK). The material selection strategy adhered strictly to a prescriptive filtering approach rather than a simple database scan. Filtering criteria were defined as limit functions, guided by the general engineering material-selection framework, and then cross-checked against biomechanical and clinical considerations reported for sternum and chest wall reconstruction, including reconstruction materials, fixation strategies, and case-based implant use [4,13,14,21,22,23,29].

Table 1 provides a detailed summary of the fundamental material properties defined for sternum implants and the corresponding threshold values. This systematic approach has enabled the selection of the most functional and biocompatible material from among the available alternatives. The table specifies boundary conditions for density and some mechanical properties for material selection. Additionally, RoHS (EU) compliant grades, toxicity ratings, medical grades (USP Class VI, ISO 10993-1) [30], and MRI compatible options are identified. RoHS (EU) monitors material compliance with the European Restriction of Hazardous Substances (RoHS) legislation. MRI compatible indicates whether a material is safe for use in a magnetic resonance environment.

These specified limit values were defined to ensure that the materials considered for sternal implant applications meet the required mechanical performance and biocompatibility expectations. The pre-screening limits were not selected arbitrarily; rather, they were established by drawing on the literature on biomaterial selection, sternum and chest wall reconstruction, and related biomedical implant applications.

The selected Young’s modulus interval was intentionally kept broad at the pre-screening stage to include both metallic and high-performance polymer-based biomaterials. In line with this literature-informed screening strategy, this range does not imply equivalence with native bone. Instead, it allows subsequent narrowing through biomechanical comparison, where the compromise between stiffness, strength, and thoracic stability can be evaluated more critically.

### 2.4. Finite Element Modeling

To compensate for the absence of physical experimental testing, finite element analysis was used as a comparative biomechanical validation tool, as supported by prior implant selection and chest wall reconstruction studies [4,26,27].

**Figure 2:**
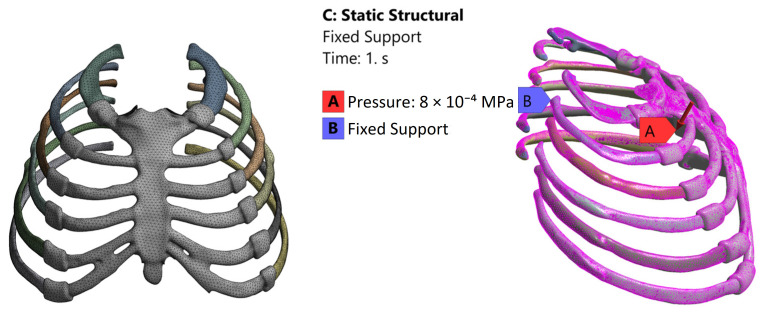
Mesh model and boundary conditions applied for FEA.

For model preparation, the implant and sternum geometries were imported into ANSYS Workbench 2024 R2 (ANSYS Inc., Canonsburg, PA, USA). A tetrahedral mesh with local refinement was generated to improve accuracy in regions expected to exhibit higher stress gradients. The final mesh consisted of 385,260 elements and 627,083 nodes. Mesh quality assessment showed an average element quality of 0.77269 and an average skewness of 0.33811, indicating acceptable overall mesh quality for comparative finite element evaluation. Boundary conditions were defined using a simplified static configuration adapted from the comparative boundary condition framework reported in [27] for the present comparative model. A single normalized pressure load together with fixed support conditions was applied for comparative finite element analysis. The applied mesh strategy and boundary conditions are illustrated in Figure 2.

Formal mesh convergence analysis, fixation hardware, and detailed bone implant interface mechanics were not included explicitly in the present framework. These choices were made to isolate the effect of bulk material behavior during preselection, but they also define the main methodological limitations of the study. In addition to mesh quality assessment, a local mesh sensitivity check was performed by refining the mesh in regions with higher stress gradients. No significant changes were observed in the overall deformation patterns or comparative trends, supporting the adequacy of the selected mesh density for the present analysis.

The current finite element model was constructed as a comparative linear elastic model. The bone domain was represented as a single homogeneous cortical bone layer with a Young’s modulus of 10.18 GPa and a Poisson’s ratio of 0.30. For comparative FEA, the Young’s modulus and Poisson’s ratio values assigned to the implant materials were taken as single representative input values derived as the midpoints of the lower and upper CES property ranges reported in Table 3. The implant materials were assigned isotropic linear elastic properties as follows: austenitic NiTi, 62.0 GPa and 0.33; PARA (50% glass fiber), 20.5 GPa and 0.33; PEEK (30% carbon fiber), 22.85 GPa and 0.44; PEEK (30% glass fiber), 9.81 GPa and 0.40; Ti-3Al-2.5V, 93.0 GPa and 0.36; Ti-6Al-4V, 113.5 GPa and 0.315; Ti-6Al-7Nb, 105.0 GPa and 0.36; Ti-12Mo-6Zr-2Fe, 76.6 GPa and 0.33; Ti-13Nb-13Zr, 78.9 GPa and 0.34; and commercially pure Ti Grade 4 (annealed), 109.5 GPa and 0.345. These values were used as the actual FEA input properties for comparative simulation. The applied surface pressure was defined as a normalized comparative surrogate load based on the simplified boundary condition approach adapted from [27]. It should therefore be interpreted as a comparative modeling condition rather than as a patient-specific physiological load.

For material assignment and comparative simulations, a representative subset of the screened candidates was modeled for comparative FEA. This subset comprised austenitic NiTi, PARA (50% glass fiber), PEEK (30% carbon fiber), PEEK (30% glass fiber), Ti-3Al-2.5V (Grade 9), Ti-6Al-4V (solution treated & aged), Ti-6Al-7Nb, Ti-12Mo-6Zr-2Fe, Ti-13Nb-13Zr (solution treated & aged), and commercially pure Ti Grade 4 (annealed). Material parameters were assigned using the actual FEA input properties summarized above, while supporting literature was used to contextualize representative material classes and biomechanical considerations [25,27,31,32,33,34]. For NiTi, the finite element model used an equivalent linear elastic representation with a Young’s modulus of 62.0 GPa and a Poisson’s ratio of 0.33. This simplification does not capture phase transformation or superelastic behavior and is therefore considered a modeling limitation.

The simulations quantified equivalent (von Mises) stress distribution, total deformation and displacement patterns, comparative load-bearing response under quasi-static loading, and a comparative stress shielding index (SSI) describing relative load sharing between the implant and the surrounding bone. In this study, SSI values closer to zero were interpreted as more balanced stress sharing; positive values indicated more implant-dominant load transfer, whereas negative values indicated a shift toward greater stress transfer in bone.

For performance evaluation, each candidate material was analyzed under an identical loading scenario to enable direct comparison. Within the assumptions of the present generic model, Ti-13Nb-13Zr showed the most balanced response among the investigated candidates because its lower modulus reduced stiffness mismatch while maintaining low deformation. Ti-6Al-7Nb also showed favorable structural performance, whereas PEEK-based systems remained more attractive for hybrid or adjunct concepts than for the main load-bearing region.

## 3. Results

Sternal implant material selection requires a balance between mechanical stability, biocompatibility, manufacturability, radiological compatibility, and long-term clinical safety. Stability and rigidity are necessary to prevent paradoxical motion and maintain chest wall integrity, whereas biocompatibility and infection resistance shape host tolerance and postoperative outcome. Flexibility and formability influence intraoperative adaptation to the defect, and the overall risk of complications also depends on soft tissue coverage and wound healing conditions [15,17,19,20].

Guided by these principles, the present study implemented a rule-based, multicriteria screening using CES software under the boundary conditions specified in Table 1. The screening returned 17 distinct candidates spanning metallic alloys and high-performance polymers (Table 2). Given that some entries belong to the same material family but differ by processing history or condition, such as annealed versus aged, a representative comparison across distinct classes was generated (Table 3) to enable an interpretable group level assessment.

In addition to the overall candidate pool, representative materials from each group were compared in Table 3 to highlight how reinforcement type, composition, and processing history influence the final property envelope within the same material family.

### 3.1. Candidate Comparison

Table 2 lists the 17 candidate materials that satisfied the predefined boundary conditions (density, stiffness window, fatigue and fracture resistance, toxicity class, RoHS compliance, MRI safety). The pool includes austenitic NiTi, fiber reinforced polymers (PARA, PEEK), commercially pure (CP) Ti grades, and alpha–beta and near beta titanium alloys (e.g., Ti-6Al-4V, Ti-6Al-7Nb, Ti-13Nb-13Zr). To enable a direct comparison across categories, Table 3 summarizes key mechanical, physical, and biological properties relevant to sternal reconstruction.

In brief, PEEK-based candidates offer low density and radiolucency, which can be advantageous for imaging and handling [24,25]. In the present comparison, however, these polymer-based candidates showed higher deformation than the titanium alloys, which suggests greater suitability for hybrid concepts or less demanding regions than for the main load-bearing part of the sternum. By contrast, the titanium candidates combined lower deformation with higher structural stiffness, which is more consistent with the demands commonly emphasized in chest wall reconstruction practice [17,19,28]. Among them, Ti-13Nb-13Zr is particularly attractive because its elastic modulus is lower than that of conventional titanium alloys and closer to bone [32,33]. Ti-6Al-7Nb remains a strong vanadium-free alternative within the broader family of biomedical titanium alloys, while Ti-13Nb-13Zr offers a lower-modulus option within the same group [35,36]. Austenitic NiTi offers high toughness and pseudoelasticity, but concerns related to surface condition, corrosion behavior, and biocompatibility still justify caution for permanent implantation [34].

Because the sternum functions as part of a compliant thoracic system rather than as a purely axial load-bearing bone, material selection should also consider the balance between structural support and physiological chest wall motion. For this reason, compliance is discussed together with strength and stiffness in the revised interpretation.

**Figure 3:**
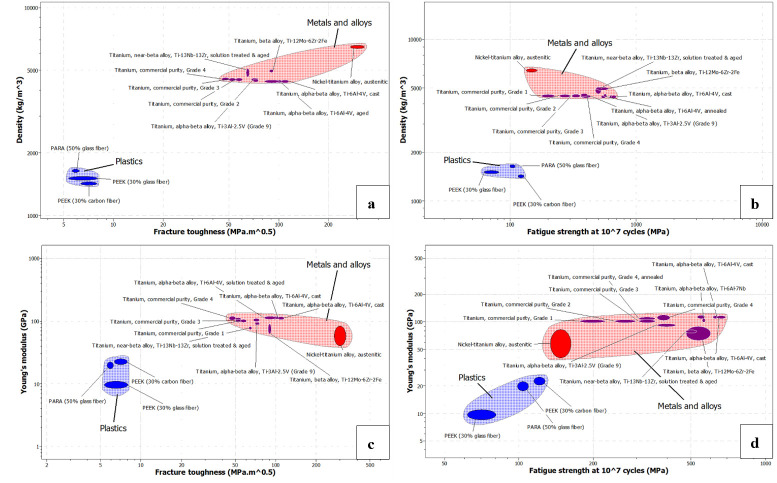
Ashby diagrams: (**a**) density vs. fracture toughness, (**b**) density vs. fatigue strength, (**c**) Young’s modulus vs. fracture toughness, and (**d**) Young’s modulus vs. fatigue strength.

To visualize these relationships, Ashby plots comparing density, fracture toughness, fatigue strength, and elastic modulus were generated (Figure 3). The plots show that titanium alloys occupy the region of higher strength and toughness, whereas high-performance polymers cluster in the lower-density region, but with lower toughness and fatigue resistance. This pattern is consistent with the current chest wall reconstruction literature, which continues to favor customized titanium solutions when structural stability is the main priority [14,18,19,28,37].

### 3.2. Finite Element Results

**Figure 4:**
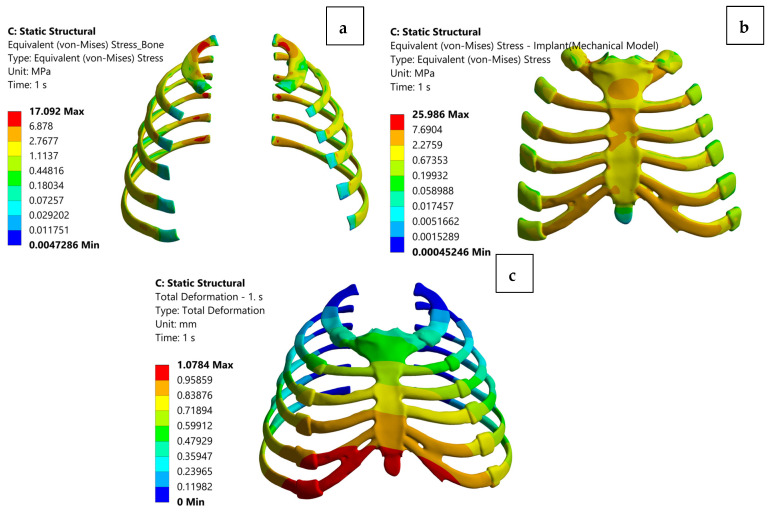
Finite element analysis results for the rib cage model with an austenitic NiTi implant: (**a**) equivalent von Mises stress distribution in bone, (**b**) equivalent von Mises stress distribution in the implant, and (**c**) total deformation of the rib cage.

To complement material screening and compensate for the absence of physical tests, finite element analysis (FEA) was used as an in silico validation method. Simulations assessed equivalent (von Mises) stress, total deformation, and bone stress under the applied comparative loading condition. Figure 4 presents an illustrative example for the rib sternum model with an austenitic NiTi implant, showing: (a) stress distribution in bone, (b) stress distribution in the implant, and (c) total deformation of the rib cage. In this representative case, the NiTi implant showed a balanced load-sharing pattern within the present comparative model. Under the current linear elastic representation, the stress state in the implant remained within the modeled response range, while the overall deformation remained within the range calculated for this comparative loading condition. These results support NiTi as a mechanically interesting candidate; however, they should not be interpreted as a direct prediction of superelastic behavior, and concerns related to surface condition, corrosion behavior, and biocompatibility still justify caution for permanent load-bearing use [34].

**Figure 5:**
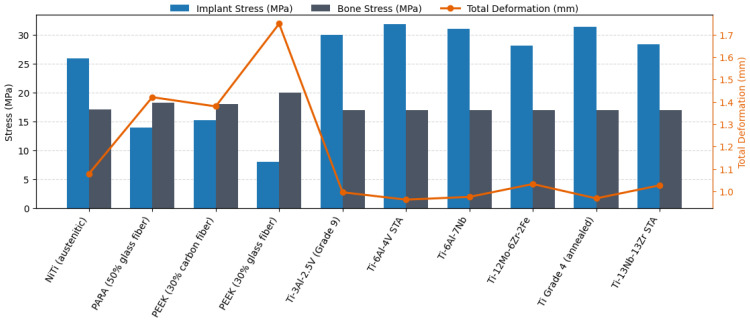
Finite element analysis results for implant stress (MPa), bone stress (MPa), and total deformation (mm).

The finite element analysis results presented in Figure 5 illustrate the distribution of implant stress, bone stress, and total deformation following implant placement. As shown in Figure 5, the von Mises stress distribution within the implant indicates that titanium alloys, particularly Ti-13Nb-13Zr and Ti-6Al-7Nb, remain within safe loading limits relative to their corresponding yield strength ranges, without exhibiting localized stress concentrations that may compromise implant integrity. Bone stress levels also remain similar across all metallic candidates (≈17 MPa), suggesting a broadly comparable load-transfer response in the bone despite differences in implant stiffness.

The total deformation profiles in Figure 5 reveal that polymer materials, especially glass fiber reinforced PEEK, exhibit higher deformation values (≈1.74 mm). In contrast, titanium alloys show substantially lower deformation ranges (≈0.96–1.03 mm), supporting their suitability for structurally stable sternal reconstruction. Ti-13Nb-13Zr, with its moderate elastic modulus, demonstrates a balanced biomechanical response, combining favorable stress distribution with controlled deformation.

Overall, the results in Figure 5 highlight that appropriate implant material selection must consider not only high mechanical strength but also load transfer characteristics, bone implant mechanical interaction, and deformation behavior under the present comparative loading condition. In this context, Ti-13Nb-13Zr emerges as the most balanced candidate due to its elastic modulus being closer to that of bone and its stable mechanical performance. Titanium alloys therefore remain the most suitable options for sternal reconstruction, offering both structural reliability and favorable biomechanical compatibility.

**Figure 6:**
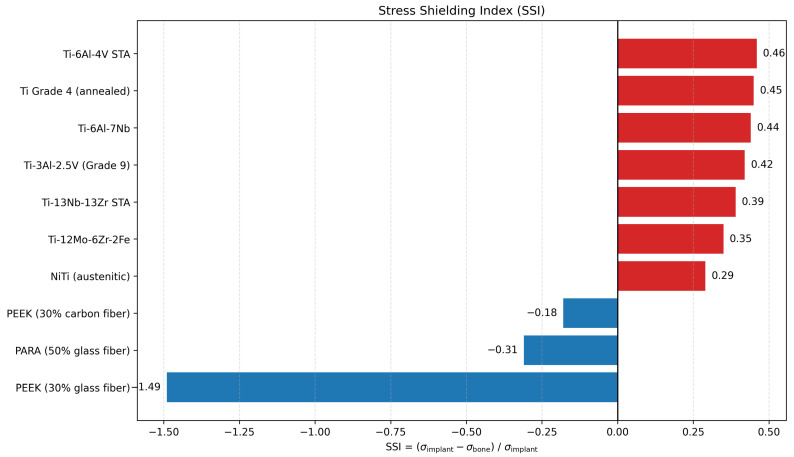
Stress shielding index (SSI) of the candidate materials. Values closer to zero indicate a more balanced stress sharing condition between the implant and the surrounding bone.

Figure 6 summarizes the SSI values for the candidate materials. In this study, values closer to zero were taken to indicate more balanced stress sharing between the implant and the surrounding bone. All metallic candidates remained on the positive side of the scale. NiTi showed the lowest SSI among the metals at 0.29, followed by Ti-12Mo-6Zr-2Fe at 0.35 and Ti-13Nb-13Zr at 0.39. The highest SSI values were observed for Ti-6Al-4V STA (0.46) and annealed Ti Grade 4 (0.45). 

The polymer-based candidates fell on the negative side, with CF PEEK at −0.18, PARA at −0.31, and GF PEEK at −1.49. In the revised interpretation, these negative values are not treated as automatically favorable. Instead, they indicate a shift of load transfer away from the implant and toward the surrounding bone, which in the case of GF PEEK is accompanied by substantially greater deformation. Accordingly, SSI was interpreted together with both implant stress and deformation rather than as a standalone ranking criterion.

## 4. Discussion

When the CES screening, Ashby diagrams, finite element results, and SSI trends are considered together, the results support a graded interpretation rather than an absolute ranking. Among the tested candidates, Ti-13Nb-13Zr provided the most balanced overall response within the assumptions of the present model because it combined relatively low deformation with a lower modulus than conventional titanium alloys. Ti-6Al-7Nb also performed well mechanically, while Ti-6Al-4V remained structurally strong but more implant-dominant in its load sharing profile. PEEK-based candidates shifted stress transfer toward bone, but this was accompanied by higher deformation, which limits their interpretation as primary load-bearing options in the central sternum. Austenitic NiTi remained mechanically interesting, yet its simplified linear elastic representation and known surface and corrosion related considerations warrant caution. Although direct experimental validation was not performed, sternum-related mechanical test development studies have described fatigue-oriented bench-top approaches that may support future validation of such constructs [38]. The predicted stress and deformation ranges are nevertheless consistent in order of magnitude with previously reported numerical studies on chest wall reconstruction and sternal implants. This agreement supports the internal consistency of the present comparative model despite its simplified assumptions. 

Ti-13Nb-13Zr appears to offer the best overall compromise in this study, since its elastic modulus is closer to that of bone, its deformation remained low, and its SSI was lower than that of the conventional titanium grades. Taken together, these findings are consistent with the broader preference for lower modulus biomedical titanium alloys [33,35,36]. Ti-6Al-7Nb also performed well mechanically in the present simulations and belongs to the group of titanium alloys already used in biomedical applications [35]; however, its higher stiffness and more positive SSI suggest less balanced load sharing than Ti-13Nb-13Zr. Ti-6Al-4V remains attractive because of its strength and long clinical history, but its higher modulus and higher SSI point to a greater tendency toward implant-dominant loading, in line with the stress shielding concerns reported for conventional titanium systems [33]. PEEK-based candidates such as CF PEEK and GF PEEK shifted stress transfer toward bone, as reflected by their negative SSI values; even so, their larger deformation values, particularly for GF PEEK, suggest within the present model that they may be more suitable for hybrid concepts or less demanding regions than for the main load-bearing part of the sternum. Austenitic NiTi showed the lowest positive SSI among the metallic candidates, which makes it mechanically interesting; nevertheless, the long-term use of nickel-containing materials still warrants caution regarding surface condition, corrosion behavior, and biocompatibility, so its role would require careful case-based justification [34].

Overall, this ordering is in line with recent clinical and biomechanical reports that continue to favor patient-specific titanium reconstruction when anatomical fit and structural stability are the main priorities [14,18,37].

It should be noted that the bone domain was simplified as a single homogeneous cortical layer, and the trabecular (cancellous) core of the sternum was not explicitly modeled. This simplification may influence local stress distributions and represents a limitation of the present approach.

From a stress shielding perspective, the SSI trend also fits the broader implant literature. Lower modulus titanium alloys are generally preferred over conventional Ti-6Al-4V because they reduce stiffness mismatch and improve load transfer [32,33,36]. The present model showed the same tendency. Ti-13Nb-13Zr gave a lower SSI than Ti-6Al-4V while keeping deformation within the low range seen for the titanium group. By contrast, the PEEK-based materials moved toward zero or into the negative range, which indicates a shift in stress transfer toward bone, but that shift came with noticeably larger deformation. This is why SSI should not be read on its own in sternal reconstruction. A material that shares stress more evenly is useful only if it also provides enough stiffness to keep the chest wall stable. This balance helps explain why titanium-based patient-specific implants still dominate current chest wall reconstruction practice [19,28]. Therefore, SSI should be interpreted as a relative indicator of load-sharing behavior rather than a direct predictor of clinical performance.

Recent clinical series have further supported customized titanium-based reconstruction for complex sternal defects, reporting encouraging perioperative and short- to mid-term outcomes for patient-specific prosthetic strategies [39,40]. At the same time, newer literature underscores that three-dimensional planning, patient-specific implant design, and continued biomechanical optimization of both metallic and polymer-based solutions are becoming central to contemporary chest wall reconstruction workflows [41,42].

Consequently, this in silico framework highlights the potential of low modulus alloys such as Ti-13Nb-13Zr for sternal reconstruction while providing decision support before physical prototyping. However, the results remain sensitive to the applied boundary conditions, generic geometry, linear elastic material assumptions, and the fully bonded representation of the bone implant interface without friction or micromotion. These findings should therefore be interpreted as pre-experimental guidance rather than as a definitive clinical prescription. To improve the clinical relevance of the framework, future studies should incorporate patient-specific imaging, patient-specific fixation strategies, mesh convergence analysis, advanced constitutive modeling, and experimental validation. 

## 5. Conclusions

This study presents an in silico framework for sternal implant material preselection that integrates anatomical reconstruction logic, rule-based CES screening, and comparative finite element analysis. Within the assumptions of the present generic model, Ti-13Nb-13Zr emerged as the most balanced candidate among the materials evaluated, while Ti-6Al-7Nb also remained a strong metallic alternative. PEEK-based materials may be more suitable for adjunct, interface, or hybrid concepts than for the primary load-bearing portion of the sternum. These findings should be interpreted as pre-experimental guidance rather than as definitive evidence of clinical superiority.

From a biomimetic perspective, the results highlight the importance of matching not only implant geometry, but also the mechanical behavior of native bone. Advances in additive manufacturing may further enable anatomically conformal and functionally graded implant designs with improved load transfer at the bone–implant interface. Future work should combine refined finite element modeling with experimental testing, surface engineering, and clinical validation to translate these computational findings into reliable chest wall reconstruction strategies.

## Figures and Tables

**Table 1:** Boundary conditions used for implant material selection.

Form	Bulk Material
Density (kg/m^3^)	≤7 × 10^3^
Young’s modulus (GPa)	10 to 120
Fatigue strength at 10^7^ cycles	≥60 MPa
Fracture toughness (MPa.m^0.5^)	≥5
RoHS (EU) compliant grades?	✓
Toxicity rating	Slightly toxic, Non-toxic
Medical grades? (USP Class VI, ISO 10993-1) [30]	✓
MRI safe	Safe

**Table 2:** The candidate materials determined by CES.

No	Candidate Material
1	Nickel–titanium alloy, austenitic
2	PARA (50% glass fiber)
3	PEEK (30% carbon fiber)
4	PEEK (30% glass fiber)
5	Titanium, alpha–beta alloy, Ti-3Al-2.5V (Grade 9)
6	Titanium, alpha–beta alloy, Ti-6Al-4V, aged
7	Titanium, alpha–beta alloy, Ti-6Al-4V, annealed
8	Titanium, alpha–beta alloy, Ti-6Al-4V, cast
9	Titanium, alpha–beta alloy, Ti-6Al-4V, solution treated and aged
10	Titanium, alpha–beta alloy, Ti-6Al-7Nb
11	Titanium, beta alloy, Ti-12Mo-6Zr-2Fe
12	Titanium, commercial purity, Grade 1
13	Titanium, commercial purity, Grade 2
14	Titanium, commercial purity, Grade 3
15	Titanium, commercial purity, Grade 4
16	Titanium, commercial purity, Grade 4, annealed
17	Titanium, near-beta alloy, Ti-13Nb-13Zr, solution treated and aged

**Table 3:** Comparison of the candidate materials.

	Nickel–Titanium Alloy, Austenitic	PARA (50% Glass Fiber)	PEEK (30% Carbon Fiber)	PEEK (30% Glass Fiber)	Ti-3Al-2.5V (Grade 9)	Ti-6Al-4V, Solution Treated and Aged	Ti-6Al-7Nb	Ti-12Mo-6Zr-2Fe	Titanium, Commercial Purity, Grade 4, Annealed	Ti-13Nb-13Zr, Solution Treated and Aged
**Density** **(kg/m^3^)**	6410–6540	1630–1660	1420–1440	1490–1540	4470–4490	4410–4450	4510–4530	4980–5000	4490–4530	4650–5100
**Young’s modulus (GPa)**	41–83	17.8–22.2	20.7–25	8.62–11	91–95	110–117	100–110	63.1–90.1	107–112	76.2–81.6
**Yield strength (elastic limit) (MPa)**	195–690	231–289	190–228	124–158	483–620	827–1070	895–905	906–1150	172–483	785–914
**Elongation (% strain)**	5–50	1.8–2.6	1–4	2–3	15–20	10–12	10–15	10–15	10–25	8–16
**Poisson’s ratio**	0.32–0.34	0.329–0.334	0.43–0.45	0.39–0.41	0.35–0.37	0.31–0.323	0.35–0.37	0.31–0.35	0.34–0.354	0.333–0.35
**Hardness–Vickers (HV)**	1230–1430	74.1–81.9	49.2–54.7	37.2–47.3	103–113	380–420	270–290	327–345	195–205	282–304
**Fatigue strength at 10^7^ cycles (MPa)**	134–162	98.8–109	115–127	61.7–80.3	363–432	613–638	559–564	477–598	306–356	475–525
**Fracture toughness (MPa.m^0.5^)**	271–328	5.6–6.19	6.34–7.89	5.34–7.93	70–75	82–100	68–75	88–92	50–55	63.7–65.5
**Toxicity rating**	Slightly toxic	Non-toxic	Non-toxic	Non-toxic	Non-toxic	Non-toxic	Non-toxic	Slightly toxic	Non-toxic	Non-toxic

## Data Availability

All data supporting the findings of this study are available within the article.
